# Potential Use of GLP-1 and GIP/GLP-1 Receptor Agonists for Respiratory Disorders: Where Are We at?

**DOI:** 10.3390/medicina60122030

**Published:** 2024-12-09

**Authors:** Miodrag Janić, Sabina Škrgat, Matevž Harlander, Mojca Lunder, Andrej Janež, Anca Pantea Stoian, Mohamed El-Tanani, Viviana Maggio, Manfredi Rizzo

**Affiliations:** 1 Department of Endocrinology, Diabetes and Metabolic Diseases, University Medical Centre Ljubljana, 1000 Ljubljana, Slovenia; mojca.lunder@kclj.si (M.L.); andrej.janez@kclj.si (A.J.); 2 Faculty of Medicine, University of Ljubljana, 1000 Ljubljana, Slovenia; sabina.skrgat@kclj.si (S.Š.); matevz.harlander@kclj.si (M.H.); 3 School of Medicine, PROMISE Department of Health Promotion Sciences Maternal and Infantile Care, Internal Medicine and Medical Specialties, University of Palermo, 90133 Palermo, Italymanfredi.rizzo@unipa.it (M.R.); 4 Department of Pulmonary Diseases and Allergy, University Medical Centre Ljubljana, 1000 Ljubljana, Slovenia; 5 Department of Diabetes, Nutrition and Metabolic Diseases, Carol Davila University of Medicine and Pharmacy, 050474 Bucharest, Romania; anca.stoian@umfcd.ro; 6 College of Pharmacy, Ras Al Khaimah Medical and Health Sciences University, Ras Al Khaimah P.O. Box 11172, United Arab Emirates; eltanani@rakmhsu.ac.ae

**Keywords:** respiratory disorders, GLP-1 receptor agonists, GIP/GLP-1 receptor agonists, asthma, COPD

## Abstract

Chronic respiratory disorders are the third leading cause of mortality globally. Consequently, there is a continuous pursuit of effective therapies beyond those currently available. The therapeutic potential of the glucagon-like peptide-1 (GLP-1) and the glucose-dependent insulinotropic polypeptide/GLP-1 (GIP/GLP-1) receptor agonists extends beyond the regulation of glycemia, including glucometabolic, cardiovascular, and renal effects, rendering them viable candidates, due to their mechanisms of action, for the possible treatment of respiratory disorders. This manuscript aims to provide a comprehensive evaluation of the evidence on potential direct (cellular) and indirect (metabolic) actions of GLP-1 and GIP/GLP-1 receptor agonists within the pulmonary systems. In addition, it examines their efficacy in addressing prevalent respiratory disorders, specifically chronic obstructive pulmonary disease (COPD), asthma, pneumonia, obstructive sleep apnea, pulmonary hypertension, lung cancer, and lung transplantation. Finally, the manuscript seeks to identify potential avenues for further focused research in this field.

## 1. Introduction

Glucagon-like peptide-1 (GLP-1) and glucose-dependent insulinotropic polypeptide/GLP-1 (GIP/GLP-1) receptor agonists are well-established in various clinical settings, including glucose control in type 2 diabetes and weight loss in people with and without diabetes [1,2]. Furthermore, clinical trials have established that GLP-1 receptor agonists, including liraglutide, dulaglutide, and semaglutide, are effective in reducing major adverse cardiovascular events (MACE) in people with type 2 diabetes [3,4,5]. The currently available GLP-1 receptor agonists are derived either from the exendin-4 molecule (exenatide) or from the human GLP-1 scaffold (liraglutide, dulaglutide, and semaglutide). Exenatide exists in short- and long-acting formulations, whereas all human GLP-1 analogs are formulated as long-acting agents. Tirzepatide represents the only available GIP/GLP-1 receptor agonist, and it is derived primarily from the human GIP hormone, which exists as a long-acting formulation [6]. Regardless of diabetes status, semaglutide contributes to a reduction in MACE and improves heart failure outcomes in those with mildly reduced and preserved ejection fractions, as well as effectively mitigating adverse renal events [3,4,7]. Beyond the effects validated in clinical trials, the GLP-1 and GIP/GLP-1 receptor agonists exhibit additional advantageous pleiotropic effects. The ability of these medications to reach supraphysiological concentrations relative to native GLP-1 and GIP, and their impact on receptors distributed throughout the body, has attracted increased attention. GLP-1 receptors are found in the endocrine and possibly exocrine pancreas, heart, adipose tissue, brain, lung, kidney, blood vessels, gastrointestinal tract, adrenal glands, spleen, eyes, and possibly bone. GIP receptors are present in the endocrine pancreas, heart, adipose tissue, brain, lung, kidney, bone, gastrointestinal tract, adrenal glands, and eyes [8].

Type 2 diabetes is a devastating disease that is increasing in prevalence. It has been associated with chronic obstructive pulmonary disease (COPD), asthma, and obstructive sleep apnea, while it is also accepted as an independent risk factor for pulmonary fibrosis [9]. The associations between diabetes and pulmonary diseases are complex, probably mediated by hyperglycemia, low-grade chronic inflammation, oxidative stress, hyperinsulinemia, endothelial dysfunction, autonomic neuropathy, and impaired mobility of the pulmonary muscles. Furthermore, chronic respiratory disorders represent a global health problem, as they are the third leading cause of death worldwide [10]. As diabetes and pulmonary diseases interconnect, it seems logical to search for a common denominator in their management. The GLP-1 and GIP/GLP-1 receptor agonists present one of the possibilities in this regard. Associations have been investigated in two meta-analyses. In the first meta-analysis of seven large randomized controlled trials that included 55,922 people with type 2 diabetes, 27,942 people were treated with the GLP-1 receptor agonist. The incidence of 12 types of respiratory diseases was evaluated, although their incidence was overall very low, the lowest for interstitial lung disease (0.02% in the GLP-1 receptor agonist group and 0.04% in the control group) and the highest for pneumonia (2.05% in the GLP-1 receptor agonist group and 2.31% in the placebo group). The study showed that, although not at statistical significance, there was a trend for a lower incidence of bronchitis, pneumonia, upper respiratory lung infection, lung squamous cell carcinoma, acute respiratory failure, asthma, chronic obstructive lung disease (COPD), pulmonary fibrosis, and lung oedema. The incidence of interstitial lung disease was higher in the group treated with the GLP-1 receptor agonist, while the effect was neutral for lung adenocarcinoma and obstructive sleep apnea. This study has a weakness with respect to the small incidence of respiratory disease [11]. However, as there is an increased incidence of pneumonia, asthma, and COPD in diabetes [12], this study reaffirms the beneficial effect of GLP-1 receptor agonists in reducing the number of these diseases. The second meta-analysis in 77,485 people from 28 randomized clinical trials showed that in people with type 2 diabetes, overweight, and obesity, treatment with GLP-1 receptor agonists was associated with a significant reduction of 14% in the relative risk, while separate medications, semaglutide, liraglutide, and dulaglutide, were associated with a reduction in the relative risk of 18%, 14%, and 18% for overall respiratory disease. These results were independent of the duration of treatment, the type of control, and the indication for GLP-1 receptor agonist treatment [13]. Therefore, the purpose of this manuscript is to review current evidence for possible actions of GLP-1 and GIP/GLP-1 receptor agonists in the lungs, as well as data on their efficacy in most common respiratory disorders, and to establish whether there is room for further dedicated research in this field. An extensive examination of the current body of literature within the relevant field was conducted utilizing accessible bibliographic databases.

## 2. Probable Mechanisms of the Beneficial Effects of GLP-1 and GIP/GLP-1 Receptor Agonists in the Lung

**Figure 1:**
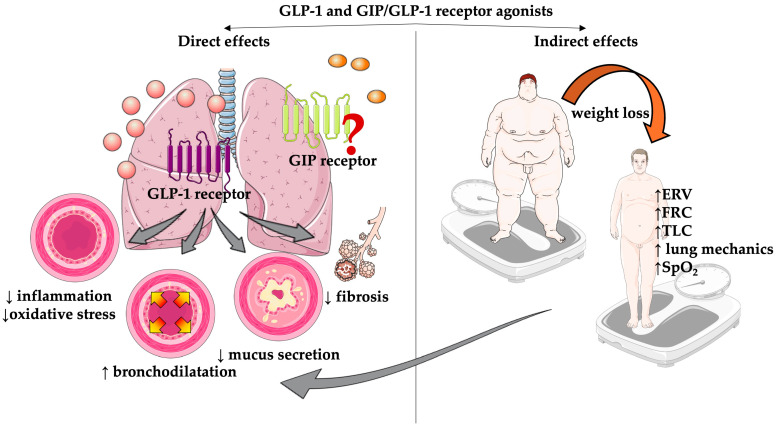
Summary of the direct and indirect effects of glucagon-like peptide-1 (GLP-1) and glucose-dependent insulinotropic polypeptide/GLP-1 (GIP/GLP-1) receptor agonists in lungs. The red question mark indicates that the effects mediated by GIP receptors in the lungs have not yet been fully understood, despite the confirmed presence of these receptors in the lungs. The grey arrow indicates that the indirect effects associated with weight loss also positively affect the direct effects achieved by the GLP-1 and GIP/GLP-1 receptor agonists in the lungs. ERV, expiratory residual volume; FRC, functional respiratory capacity; TLC, total lung capacity; SpO_2_, blood oxygenation.

Both GLP-1 and GIP receptors can be found in the lung. These receptors are located within multiple lung structures (alveoli pneumocytes, ciliated and club cells, smooth muscle cells of the septa, and the airways and vascular smooth muscles as well as immune cells) and their expression levels appear to be the highest in the lung compared to other organs outside the pancreas [8,14]. Furthermore, the concentration of GLP-1 in the bronchoalveolar fluid is higher than in the serum, thus signaling its important function in the lungs [15]. The evidence on the distribution and effects of GIP receptors in the lungs is limited, and further research is necessary to elucidate their role in the pulmonary immune response [16]. The summary of probable direct and indirect effects mediated by the GLP-1 and GIP/GLP-1 receptor agonists in the lungs is presented in Figure 1.

### 2.1. Direct Effects

The direct effects of GLP-1 receptor agonists and probably GIP/GLP-1 receptor agonists in the lungs are mediated primarily through the GLP-1 receptors, namely anti-inflammatory and antioxidant, bronchodilator, mucus-producing, and antifibrotic mechanisms (Figure 1). GLP-1 receptor agonists can modulate immune responses by influencing the signaling of immune cells and changing the signaling environment from pro-inflammatory to anti-inflammatory. This is coupled with a reduced oxidative stress, and these effects are beyond the glucose-lowering effects of these drugs.

**Figure 2:**
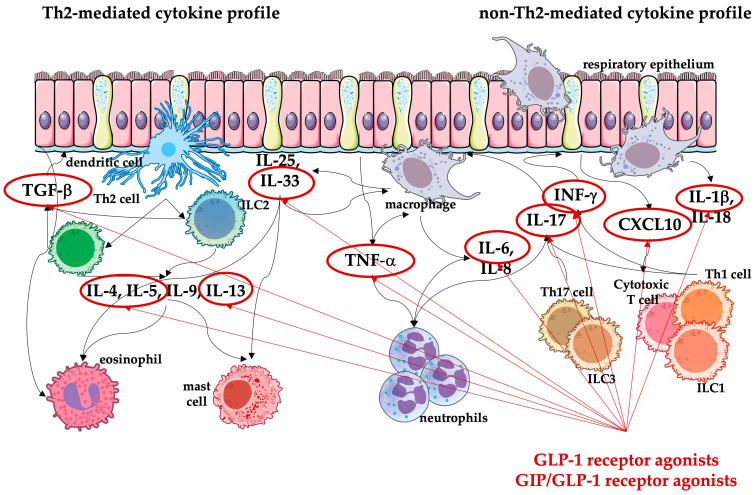
Main cytokine profiles attenuated by glucagon-like peptide-1 (GLP-1) and glucose-dependent insulinotropic polypeptide/GLP-1 (GIP/GLP-1) receptor agonists. The T helper cells 2 (Th2)-mediated cytokine profile is characteristic mainly for asthma and to a lesser degree for chronic obstructive pulmonary disease (COPD), while the non-Th2 mediated cytokine profile can be found in obesity-related asthma and COPD [23,24]. The red arrows show the cytokines that are affected by the GLP-1 and GIP/GLP-1 receptor agonists. TGF, transforming growth factor; IL, interleukin; TNF, tumor necrosis factor; INF, interferon; CXCL, C-X-C motif chemokine ligand; Th cell, T helper cell; ILC, innate lymphoid cell.

Many immune cells, including macrophages, monocytes, eosinophils, and lymphocytes, express GLP-1 receptors. By binding to them, GLP-1 receptor agonists act in different ways. Intracellular signaling is mediated by adenylate cyclase (AC), which, through two subsequent cascades, further transmits the signal. The first cascade involves cyclic adenosine monophosphate (cAMP)-calcium that results in stimulation of the energy-sensing AMP-activated protein kinase (AMPK). Second, it involves the stimulation of protein kinase A (PKA). These both lead to reduced expression of the nuclear factor kappa B (NF-κB) and increased expression of signal transducer and activator of transcription 1 (STAT1) [17]. This is also achieved directly through the GLP-1 receptor-mediated activation of the extracellular signal-regulated kinase 1/2 (ERK1/2) pathway. Consequently, activation of these cascades leads to a reduction of the pro-inflammatory cytokine release, including tumor necrosis factor alpha (TNF-α), interleukin 6 (IL-6), IL-1β, Il-17, IL-2, and interferon γ (INF-γ), the so called non-T helper cells 2 (Th2)-mediated cytokine profile (Figure 2), and reduction in adhesion molecule release including vascular cell adhesion protein 1 (VCAM-1), intercellular adhesion molecule 1 (ICAM-1), and E-selectin. Furthermore, chemokine release is also reduced, including the C-X-C motif chemokine ligand 10 (CXCL10) and the monocyte chemoattractant protein-1 (MCP-1). Expression of IL-1β is additionally suppressed through GLP-1 receptor mediated inhibition of the protein kinase C (PKC) cascade. On the other hand, the production of anti-inflammatory cytokines, such as IL-10, increases in addition to mannose receptor-1 (MRC-1) and the release of arginine-1 (Arg-1), prostaglandin E2 (PGE2), and cyclooxygenase 2 (COX2) [18,19,20]. Collectively, these mechanisms lead to attenuation of inflammation.

Airway smooth muscle cells also express GLP-1 receptors. Signal transduction is mediated through the increase in adenosine-triphosphate-binding cassette transporter A1 (ABCA1) and decrease in IL-6, IL-8, TNF-α, as well as granulocyte and macrophage colony-stimulating factor (GM-CSF). The net effect is reduced proliferation and migration of smooth muscle cells in the airways, as well as their reduced secretion of inflammatory mediators [20].

GLP-1 receptor agonists also inhibit the formation of asymmetric dimethylarginine (ADMA) by modulating the advanced glycation end products (AGE) and their interaction with receptor for the AGE (RAGE), also through the PKA and blunting of NF-κB, thus suppressing the expression of the arginine methyltransferase-1 protein and inhibiting the production of reactive oxygen species. Administration of GLP-1 receptor agonists may also further inhibit dysregulated autophagy and inflammation induced by reactive oxygen species. Even more, endothelial nitric oxide (eNOS) synthesis, which is inhibited by ADMA, is thus augmented because of NF-κB and ADMA suppression [17,19]. Alternative immunomodulatory mechanisms of GLP-1 receptor agonists may encompass the modification of T cell functionality by diminishing the expression of CD28 and CD86, alongside the reduction of tissue factor (TF) and plasminogen activator inhibitor (PAI-1) production [16]. The antioxidative effects of GLP-1 receptor agonists can also be facilitated by the attenuation of lipid peroxidation, suppression of mitochondrial autophagic signaling pathways, and reduction in alpha-synuclein synthesis, which collectively lead to the inhibition of apoptosis and normalization of mitochondrial dynamic imbalance. Furthermore, the induction and upregulation of antioxidant enzymes, specifically superoxide dismutase and glutathione peroxidase, coupled with the downregulation of nicotinamide adenine dinucleotide phosphate (NADPH) oxidase and malondialdehyde levels, may represent a mechanism through which GLP-1 receptor agonists exert their antioxidative effects [20].

It is important to acknowledge that the mechanisms associated with GLP-1 receptor agonists, together with supplementary protein synthesis pathways within the smooth muscle cells of the airways, endothelial cells, and immune cells, may contribute to an imbalance in protease-antiprotease equilibrium. This occurs in addition to the indirect mechanisms that promote the synthesis of α1 antitrypsin in the liver. These processes result in the inhibition of the destruction of the alveolar septa and the development of emphysema [20].

Several studies indicate that GLP-1 receptor agonists may possess bronchodilator effects that can be facilitated through transduction pathways analogous to anti-inflammatory mechanisms. The activation of the cAMP/PKA, cAMP/guanine nucleotide exchange factor (GEF), and phosphatidylinositol-3 kinase (PI3)/PKC pathways by the GLP-1 receptor agonist in smooth muscle cells of the airways collectively facilitates the relaxation of these cells. Within the airway epithelium, specifically in goblet and airway epithelial cells, the agonism of GLP-1 receptors results in reduced expression of IL-33 and thymic stromal lymphopoietin (TSLP), which subsequently suppress group 2 innate lymphoid cells (ILC2), Th2 cells, and eosinophils from releasing IL-4, IL-5, and IL-13, the so-called Th2-mediated cytokine profile. This cascade culminates in decreased mucus secretion and increased surfactant production, complemented by thyroid transcription factor 1 (TTF-1) signaling pathway [10,17,21].

There is evidence suggesting that GLP-1 receptor agonists may possess antifibrotic properties, making them potential candidates for attenuating the progression of pulmonary fibrosis. In this context, IL-1β is recognized for its role in mediating extracellular matrix formation directly or through transforming growth factor β1 (TGF-β1). The latter, primarily derived from alveolar macrophages, is responsible for the differentiation of fibroblasts into myofibroblasts and the induction of fibrosis-related gene expression. Consequently, suppression of IL-1β via GLP-1 receptor mediation, particularly through the pathway involving PKC-mediated inhibition of the nucleotide-binding domain, leucine-rich-containing family, pyrin-domain-containing-3 (NLRP3) inflammasome, can further contribute to reducing the formation of a profibrotic environment. The antifibrotic effects are further facilitated by the altered interaction between the glycolytic pathway driven by 6-phosphofructo-2-kinase/fructose-2,6-biphosphatase 3 (PFKFB3) and the NLRP3 inflammasome, along with the subsequent inhibition of lactate-mediated histone lactylation [22].

### 2.2. Indirect Effects

The indirect effects of the use of GLP-1 and GIP/GLP-1 receptor agonists on the lung are probably mediated by weight loss and improved lung mechanics. Data from weight loss studies suggest an impact on lung function. The benefits of weight loss in overweight/obese individuals are reflected in increased expiratory residual volume (ERV), functional respiratory capacity (FRC), total lung capacity (TLC), gas exchange, and consequent increase in blood oxygenation in addition to improvement in respiratory muscle strength and dyspnea [25]. Furthermore, weight loss is associated with a substantial decrease in adipose tissue generated pro-inflammatory mediators, probably enhancing the direct anti-inflammatory and antioxidant effects of these drugs [19].

Semaglutide at a dose of 2.4 mg is currently the most effective GLP-1 receptor agonist for weight reduction. In a meta-analysis involving 3087 obese individuals without diabetes, it resulted in an average weight loss difference of −12.3 kg (−12.1%) compared to a placebo for an average duration of 77 weeks. Side effects were mostly gastrointestinal in the semaglutide treated groups, but showed only mild to moderate severity and did not require discontinuation of treatment [26]. There exists biological plausibility for an association between GLP-1 receptor agonists and the development of medullary thyroid carcinomas in rodent models, as well as the formation of nonlethal thyroid C-cell tumors. Such findings were similar in two-year carcinogenicity studies involving semaglutide administered to rats and mice [27]. However, recent systematic reviews of the available evidence have indicated that there are no significant associations between semaglutide administration and thyroid cancer risk, with a reported incidence of less than 1% among patients treated with semaglutide. Furthermore, thyroid cancer has been shown to exhibit an association with overweight and obesity, implying that the population targeted for treatment with these drugs may have an increased predisposition to thyroid cancer development [28].

A pooled analysis of tirzepatide, currently the only GIP/GLP-1 receptor agonist, in 4036 participants regardless of diabetes status, for a treatment duration of 12 to 72 weeks, showed a significant weight loss of mean difference of −7.7 kg (−8.1%), −11.6 kg (−11.9%), and −11.8 kg (−12.4%) compared to placebo for doses of 5, 10, and 15 mg, respectively. Adverse events were more common in the tirzepatide-treated groups, presenting as nausea, vomiting, and diarrhea [29]. In the SURMOUNT-1 trial, 2539 people with overweight (body mass index (BMI) ≥ 27 kg/m^2^ and one obesity related complication) and obesity BMI ≥ 30 kg/m^2^ were treated with tirzepatide 5, 10, or 15 mg or placebo for 72 weeks. The mean percentage of weight reduction at 72 weeks ranged from −15.0%, −19.5%, and −20.9% for tirzepatide 5, 10, and 15 mg compared to −3.1% in the placebo group. Overall, 85% of the participants achieved more than 5% weight loss with a dose of 5 mg, while 57% of the participants achieved more than 20% weight loss at 15 mg. The adverse events that led to discontinuation of therapy were 4.3%, 7.1%, and 6.2% for 5, 10, and 15 mg of tirzepatide, respectively, and 2.6% in the placebo-treated group [30]. Data indicate a substantial impact on weight reduction, suggesting a promising ancillary role for GLP-1 and GIP/GLP-1 receptor agonists, which could contribute to an indirect improvement in lung function.

## 3. GLP-1 and GIP/GLP-1 Receptor Agonists and Common Pulmonary Diseases

### 3.1. Chronic Obstructive Pulmonary Disease

Chronic obstructive pulmonary disease (COPD) is characterized by airway obstruction that is poorly reversible. Chronic inflammation appears to be the primary cause. There may be obstruction of small airways leading to chronic obstructive bronchitis and destruction of the alveolar walls, known as emphysema, resulting in air trapping, dyspnea, and diminished physical capacity. It is strongly associated with smoking and air pollution. Smoking cessation is the only intervention known to reverse the progression of the disease. According to the World Health Organization (WHO), COPD ranks seventh in the leading cause of poor health and third in the leading cause of mortality globally [31]. COPD can be accompanied by diabetes, and patients with both conditions are more likely to experience severe exacerbations and death compared to COPD patients without diabetes [20]. As this is the case, there is a need for additional treatment that would lessen the burden of each disease on the others.

Potential common strategy could be based on the use of GLP-1 and GIP/GLP-1 receptor agonists. Data on the effects of GLP-1 receptor agonists have been published in patients with COPD, while no data on GIP/GLP-1 receptor agonists have been found. In one study, 1642 patients with type 2 diabetes and COPD from the US health system participated in a retrospective, observational, health record-based study. The active comparator was implemented, with a new user design, with users of the GLP-1 receptor agonist defined as the reference group. Fewer unadjusted COPD exacerbations were recorded in GLP-1 receptor users compared to significantly higher adjusted exacerbation rates with dipeptidyl peptidase 4 (DPP-4) inhibitor users and sulfonylurea users. Users of the GLP-1 receptor agonists were also less likely to experience severe exacerbations. No statistically significant differences were recorded between users of the GLP-1 receptor agonist and users of the sodium-glucose cotransporter 2 (SGLT-2) inhibitor [32]. These results were not stratified according to BMI, as this could also lead to fewer exacerbations and the possible impact due to weight loss was not considered. Furthermore, the impact of COPD overlap syndrome was not considered [33]. However, this gives a signal of a favorable effect of GLP-1 receptor agonist therapy in the COPD population, as was also previously shown in a similar study in the UK, where a 30% reduction per 100 person years of severe exacerbations was recorded in GLP-1 receptor agonist users compared to sulfonylurea users, as well as a 37% reduction per 100 person years of moderate exacerbations. There was a similar finding for the users of SGLT-2 inhibitor, but only for severe exacerbations [34]. Furthermore, among 8060 Taiwanese people with type 2 diabetes and COPD, users of the GLP-1 receptor agonist with a median follow-up duration of 2.51 years, the risk of pulmonary complication was significantly lower than in non-users. Users of the GLP-1 receptor agonists were significantly less likely to need non-invasive positive pressure ventilation, invasive mechanical ventilation, and to have bacterial pneumonia than nonusers. This observation was documented among non-obese individuals, while no notable reduction in either outcome was observed for obese individuals; this may suggest that these findings are not attributable to weight reduction. Additionally, a significantly lower risk of mortality and cardiovascular events was recorded in the users group. A longer duration of GLP-1 receptor agonist use was also associated with a lower risk of invasive mechanical ventilation, bacterial pneumonia, mortality, and cardiovascular events [35]. Only one prospective study evaluated the impact of liraglutide versus placebo in 30 people with obesity and COPD at different times on lung function tests, 6 min walk test, and responses to a questionnaire on the clinical impact of COPD (COPD assessment test—CAT score). After 40 weeks of follow-up, body weight loss was significant in users of liraglutide, forced vital capacity increased significantly, as did carbon monoxide diffusion capacity, while the CAT score improved. Other lung parameters did not reach statistical significance compared to the placebo group, but this opens the way for the preferred treatment with GLP-1 receptor agonists in this clinical setting [36].

Peripheral blood mononuclear cells (PBMC) isolated from COPD patients showed defective signaling of the GLP-1 receptor compared to subjects without COPD. The GLP-1 receptor dysfunction was characterized by decreased INF-γ expression that led to the defective T-cell functioning. When PBMC were incubated in liraglutide, the GLP-1 receptor signaling was upregulated, followed by increased INF-γ production and downregulated programmed cell death protein 1 expression on T-cells [37]. Furthermore, in female C57BL/6 mice with induced metabolic syndrome and emphysema, treatment with the GLP-1 receptor agonist has been shown to reduce the area of emphysema, as well as to increase the number of CD31+ endothelial cells, probably through reduced inflammation and positive effects on lipid and glucose metabolism. The authors propose the creation of the GLP-1-based drug focused on epithelial regeneration, as endothelial progenitor cells have been shown to be a pathogenetic and disease marker in this setting [38]. This is also in accordance with indirect mechanisms that promote the GLP-1 receptor agonists-mediated synthesis of α1 antitrypsin in the liver, as mentioned earlier [20]. Balk-Møller et al. showed improvement in lung function in COPD mice treated with the GLP-1 receptor agonist. They recorded less inflammation, but not less emphysema, compared to the study described above. Furthermore, mice treated with the GLP-1 receptor agonist had higher expression of atrial natriuretic peptide and decreased endothelin-1 expression, leading to a more bronchodilator environment [39]. Additionally, in mice, exendin-4 reinstated forkhead box protein A2 (FOXA2) expression lessened mucin production in airway cells affected by COPD and decreased both mucin and *P. aeruginosa* load in the lungs of mice, showing potential protective effect in COPD [40]. Similar findings for lixisenatide have been shown in 16 human bronchial epithelial cells (16HBE), where it reduced lipopolysaccharide-induced expression of mucin and inflammation by regulation of nuclear factor erythroid 2 related factor 2 (Nrf2) [41]. These mechanisms, shown in non-clinical environments, seem to translate into the clinical setting, as data from human trials show the positive impact of GLP-1 receptor agonists on favorable outcomes in patients with COPD. 

### 3.2. Asthma

Asthma is a condition that involves persistent inflammation and variable structural changes in the large and small airways. It presents with a variety of symptoms, including wheezing, difficulty breathing, chest tightness, and coughing, which can change in severity and over time. The course of the disease can vary, and, in some patients, it can quickly lead to reduced lung function. Often associated with allergic diathesis, it tends to occur less frequently in association with allergies in adults. It is often accompanied by multiorgan allergies and non-allergic health problems such as obesity, gastroesophageal reflux disease, and mental health disorders. Affecting more than 4% of the world’s population, it is among the most common chronic diseases [42]. Asthma is currently treated with inhalator glucocorticoids, long-acting beta agonists, leukotriene receptor antagonists, and long-acting antimuscarinic agents advancing to biologic therapies, particularly for the high-T2 (characterized by the subpopulation of CD4+ T cells—Th2 cells secreting the IL-4, IL-5, IL-13) resistant asthma phenotype. However, treatment gaps remain, as, despite therapy, some patients still have insufficiently controlled asthma. When asthma is associated with obesity and insulin resistance, the low T2 phenotype is more prevalent and is often resistant to targeted therapy [43,44]. This treatment gap, particularly in people with obesity and insulin resistance, could be at least partially overcome with GLP-1 and GIP/GLP-1 receptor agonists, as this clinical setting calls for weight loss (indirect anti-inflammatory action) and a potential direct anti-inflammatory action of these medications to reduce asthma exacerbations, possibly through some of the described mechanisms. 

Khan et al. report, in 7 adherent patients with type 2 diabetes and asthma treated with liraglutide for 52 weeks, an improvement in glucometabolic parameters, as well as asthma symptoms and exacerbations, since no subject adherent to liraglutide had a significant deterioration of asthma. This was not related to changes in dose or the addition of other asthma control drugs. Two patients in that cohort dropped out due to drug-related side effects, with one of them experiencing a non-fatal severe exacerbation [45]. Foer et al. followed this with the publication of research that compared the rates of asthma exacerbations and symptoms in people with type 2 diabetes using a GLP-1 receptor agonist with those using other antidiabetic drugs (SGLT-2 inhibitors, DPP-4 inhibitors, sulfonylureas, or basal insulin) to intensify the treatment of diabetes. The study was retrospective, using a new user-based health record with an active comparator, calculating propensity scores for the use of GLP-1 receptor agonists and the use of non-GLP-1 receptor agonists. They report, after adjusting for propensity scores and other covariates, that 6 months after initiation, asthma exacerbations were lower in people who started using the GLP-1 receptor agonist compared to all other drug users; the same trend was reported for asthma symptoms. The findings remained consistent even after adjustment for variations in BMI and glucose control, indicating that these associations do not depend solely on weight or glucose management. These effects were even more pronounced in patients with moderate and severe asthma. This study suggested that GLP-1 receptor agonists could be a novel solution to manage asthma-associated metabolic dysfunction [46]. Critics of this study suggest that a comparison with metformin, previously shown to improve asthma outcomes, would be necessary to further reveal the efficacy of GLP-1 receptor agonist treatment in this setting, as well as some statistical/methodical issues [47]. However, this study encouraged further research in this field. In 2024, the investigation of new antidiabetic therapies and metformin was reported on asthma exacerbations in people with asthma and type 2 diabetes. Kimura et al. report that in the Japanese national administrative database, among 137,173 new oral antidiabetic drugs users with history of asthma, people treated with GLP-1 receptor agonists had a hazard ratio of 1.14 (95% CI: 1.01–1.28) for exacerbations requiring systemic glucocorticoid therapy compared to metformin [48]. In a recent meta-analysis of 39 randomized controlled trials involving people with obesity and/or type 2 diabetes, a trend towards a reduction in the risk of asthma was recorded in users of GLP-1 receptor agonists compared to non-users. This was significant only with light molecular weight GLP-1 receptor agonists (efpeglenatide, exenatide, liraglutide, semaglutide) [49]. The most recent study used data from the UK Clinical Practice Research Datalink Aurum (2004–2020) that included new metformin users with type 2 diabetes. The findings indicate that metformin was associated with a 32% reduction in asthma attacks as reported by the patients and a 24% reduction determined by the inverse probability weighting methodologies. These results remained unaffected by variations in HbA1c, BMI, blood eosinophil counts, or severity of asthma. Furthermore, the adjunctive use of GLP-1 receptor agonists contributed to an additional 40% reduction in the relative rates of asthma attacks. Both metformin and GLP-1 receptor agonists were effective in reducing asthma attacks, independently of glucose and weight management [50].

However, the association between GLP-1 and GIP/GLP-1 receptor agonists and asthma is not unambiguous. Among 159,705 patients in 19 randomized controlled trials, data from 218 asthma patients indicated that SGLT-2 inhibitors were associated with a markedly reduced risk of asthma, with an odds ratio of 0.59 (95% CI: 0.38–0.93). In contrast, GLP-1 receptor agonists and DPP-4 inhibitors did not demonstrate a significant impact on asthma risk [51]. Recently, data from the US Food and Drug Administration (FDA) adverse event reporting system (FAERS) revealed that in the real world, particularly exenatide, liraglutide, and semaglutide were associated with increased rates of asthma and asthma-like events (for the latter two, the association was significant). Furthermore, incidents and deaths related to asthma were reported, and exenatide was responsible for the highest share of deaths. The cause of the finding could be due to multiple factors. It may be due to the spontaneous nature of the FAERS reporting mechanism, which operates without medical review, allowing for potential misclassification of cases or the appearance of reporting bias. Furthermore, the approximate incidence of adverse events was derived from signal strength rather than exact figures. From a mechanistic perspective, the increased incidence of respiratory-related adverse events associated with exenatide can be attributed to its derivation from nonhuman GLP-1, with which it shares only 50% structural homology, potentially leading to increased immunogenicity. Furthermore, the patient populations prescribed various GLP-1 receptor agonists differ, possibly influencing the risk of asthma-related adverse events (considering factors such as age, comorbidities, preexisting respiratory conditions, smoking status, genetic predisposition). On the contrary, there were fewer respiratory and asthma-like events with tirzepatide, a dual agonist of the GIP and GLP-1 receptors. This observation may reflect an additional or synergistic anti-inflammatory effect attributed to GIP receptor agonism alongside GLP-1 receptor agonism. The authors also admit that the findings may be mainly statistical rather than causal. However, these findings require careful and cautious interpretation. It is important to remember that FAERS data can provide valuable information on the respiratory safety profile of GLP-1 receptor agonists [52].

From a mechanistic point of view, GLP-1 receptor agonists were shown to reduce airway inflammation through the NF-κB PKA-dependent inflammation pathway. They also decrease the release of IL-33 through which inflammatory Th2 are reduced and IL-5 and 13 are released, leading to a reduced number of eosinophils and reduced goblet metaplasia and mucus production (Figure 2) [53]. This is supported by research conducted on purified human neutrophils and eosinophils from healthy people and asthmatics. The expression of GLP-1 receptors in eosinophils in asthma patients was significantly lower than in healthy people’s eosinophils, while the number of GLP-1 receptors in neutrophils was the same in both populations. A lipopolysaccharide challenge was carried out and significantly less surface activation markers of eosinophils were present in eosinophils treated with the GLP-1 receptor agonist, and their production of IL-4, 8, and 13, but not IL-5, decreased. This leads to the conclusion that there could be a direct effect of GLP-1 receptor agonists on eosinophils implicated in asthma exacerbation [54]. Furthermore, in obese asthma mice, the GLP-1 receptor agonist liraglutide was shown to reduce airway hyperresponsiveness and eosinophilic airway inflammation, which were associated with significant weight loss. In bronchoalveolar lavage, there was a reduction in IL-4, 5, and 33. In peribronchial tissue, a reduction in inflammation was also detected through the receptor protein 3 nucleotide oligomerization domain (NLPR3) inflammasome and IL-1β (Figure 2) [55]. Although the investigation using human isolated LPS-stimulated eosinophils did not demonstrate a reduction in IL-5, the obese asthma mouse model exhibited a decrease in IL-5 levels in bronchoalveolar lavage. The observed discrepancies are probably attributable to the different models used in their evaluation, suggesting that eosinophils are not the sole source of IL-5 production. Furthermore, the obese asthma model indicates that treatment with GLP-1 receptor agonists results in a cumulative attenuation of IL-5 production from all sources.

Similar findings have been reported for tirzepatide, showing a significant reduction in body weight, as well as glucose control and a reduction in leptin levels in the obese polygenic mouse model compared to semaglutide, GIP agonist alone, or vehicle. This was accompanied by reduction of Th2 inflammation and activation of ILC2 in tirzepatide treated mice lung compared to vehicle. There was also a reduction in IL-5, 13, and the number of Th2 cells in the lungs compared to semaglutide treatment, but with respect to IL-33, semaglutide was more powerful as its release is mediated particularly through the agonism of the GLP-1 receptor in which tirzepatide has less affinity. These findings support the fact that tirzepatide reduces allergic inflammation in the aeroallergen-induced model of asthma in obese mice [56]. In summary, GLP-1 (and possibly GIP/GLP-1) receptor agonists could improve asthma outcomes through weight reduction, decreased airway inflammation and mucus secretion, decreased type 2 inflammation signaling, reduction in IL-4, 5, 13, and 33 secretion, reduced mast cell activity, as well as increases in surfactant production and smooth muscle relaxation [57].

### 3.3. Pneumonia

Pneumonia represents a common respiratory disease that affects the alveoli and distal bronchial structures of the lungs. It is contracted primarily within the community (community-acquired) or within hospital settings (hospital-acquired), where it may also be associated with mechanical ventilation. Aspiration pneumonia accounts for 5% to 15% of community-acquired pneumonia cases, although its prevalence in hospital settings remains undetermined. The people most frequently affected are children under the age of 5 and adults over the age of 70 years, and these demographic groups also exhibit the highest mortality rates. In 2019, more than 2.5 million people were estimated to have succumbed to lower respiratory tract infections, half of these fatalities occurring in people aged 70 years or older. The etiology of pneumonia can be categorized as bacterial or viral. *Streptococcus pneumoniae* is the main causative microorganism for community-acquired pneumonia, whereas *Staphylococcus aureus* predominantly accounts for hospital-acquired cases [58,59]. The most prevalent viral pathogens responsible for pneumonia are human rhinovirus and influenza virus; recently, severe acute respiratory syndrome coronavirus 2 (SARS-CoV-2) has emerged as a significant etiological factor [58]. Individuals with diabetes exhibit an increased susceptibility to infections compared to those without diabetes, regardless of comorbid conditions and other confounding factors. People with diabetes face a two to four-fold increased risk of hospitalization due to infections, along with a 1.5-fold elevated risk of infection in outpatient settings. This pattern was particularly apparent during the COVID-19 pandemic. Diabetes is associated with more severe infection outcomes, as demonstrated by a two-fold increase in the mortality rate from COVID-19 [60].

In research carried out by Brunetti et al., they examined data from nearly 30,000 individuals, of which 705 were admitted to hospitals for community-acquired pneumonia during an average follow-up period of 1.7 years. Their findings revealed that users of SGLT-2 inhibitors faced a markedly reduced risk of hospitalization for community-acquired pneumonia compared to those using DPP-4 inhibitors. When beneficiaries were evaluated against GLP-1 receptor agonist users, no variations in risk were identified [61].

A network meta-analysis comprising 31 studies with nearly 3.7 million participants diagnosed with type 2 diabetes indicated that the incidence of adverse outcomes related to COVID-19 was the least frequent among those given SGLT-2 inhibitors, exhibiting a probability of 6% prior to their diagnosis of COVID-19. Furthermore, the use of GLP-1 receptor agonists and metformin was associated with a reduction in adverse COVID-19 outcomes compared to other antidiabetic medications, with GLP-1 receptor agonists showing a probability of 25% and metformin a probability of 28% [62]. A comprehensive meta-analysis conducted by Song et al. demonstrated that GLP-1 receptor agonists, along with SGLT-2 inhibitors and metformin, had a favorable impact on COVID-19 outcomes. The study revealed that GLP-1 receptor agonists were correlated with a 27% reduction in mortality, a 16% decrease in the risk of severe disease, and a 14% decrease in the probability of hospitalization for COVID-19 compared to insulin. The influence of various glucose-lowering medications on COVID-19 outcomes may have been moderated by their utilization in hospital settings versus nonhospital environments, their administration among elderly versus younger populations, and by geographical variations [63]. It is interesting to observe that during COVID-19, there was a notable increase in IL-6 levels, along with a marked increase in GLP-1 and procalcitonin levels at the time of admission and within 5 to 6 days after hospitalization in patients who succumbed to the disease, compared to those who survived. This phenomenon was discernible in both individuals with diabetes and without diabetes. However, the intensity of the pro-inflammatory/GLP-1 response was modulated by the presence of type 2 diabetes. Furthermore, hypoxemia specifically attenuated the GLP-1 response in people with type 2 diabetes who experienced bilateral lung damage. The authors observed that the increase in GLP-1 and procalcitonin levels indicated a bacterial infection, but the efficacy of GLP-1 receptor agonists in this particular context was not evaluated [64].

Direct application of tirzepatide in patients with COVID-19 has not yet been researched. However, potential mechanisms for reducing complications have been proposed. Tirzepatide can potentially decrease inflammation in COVID-19 cases, and its effects on weight reduction and the reduction of body fat can mitigate the severity of COVID-19. Furthermore, tirzepatide, similar to GLP-1 receptor agonists, is believed to alleviate COVID-19-induced alterations in the intestinal microbiota, thus decreasing intestinal inflammation and systemic complications in people with type 2 diabetes or obesity. Furthermore, through GIP agonism, it may inhibit the expression of IL-1β, IL-6, MCP-1, chemokines, and TNF-α [65].

A study that investigated the impact of GLP-1 receptor agonists on infectious (influenza) and non-infectious (bleomycin-induced) lung inflammation in wild-type and GLP-1 receptor knockout mice revealed that in the absence of the GLP-1 receptor, bleomycin-induced lung injury was less severe, while activation of the GLP-1 receptor was correlated with increased pulmonary inflammation mediated through the sympathetic nervous system. Administration of liraglutide led to a reduction in pathogen load in influenza-infected mice and increased survival rates [66].

In murine models of pneumonia-induced sepsis, liraglutide has been shown to significantly reduce levels of pro-inflammatory cytokines, such as TNF-α and IL-6, in the pulmonary system compared to septic control cohorts. Additionally, liraglutide significantly promoted the expression and secretion of pulmonary surfactant proteins, along with the secretion of phospholipids. These effects culminated in higher survival rates and attenuated pulmonary inflammation and damage within this specific model. GLP-1 receptor agonists have been observed to strengthen host defense mechanisms and facilitate alveolar respiratory function in the presence of acute lung injury [67].

Not unimportant, in the perioperative period, the use of GLP-1 and GIP/GLP-1 receptor agonists may be associated with an increased risk of regurgitation and aspiration of gastric contents into the lungs during induction of general anesthesia due to delayed emptying and retention of gastric contents. This is a rare but serious complication with an incidence of 1 in 3000 to 4000 elective procedures [68]. Pneumonitis, aspiration pneumonia, and other lung injuries are associated with increased morbidity and are the most common cause of anesthesia-related mortality. The greatest risk factor for them is the presence of food or fluid in the stomach immediately before the procedure [68,69]. Recommendations for the perioperative management of patients have not previously considered special measures for patients treated with GLP-1 and GIP/GLP-1 receptor agonists. However, there have been increasing reports of clinical cases of regurgitation and/or aspiration of gastric contents during induction of general anesthesia and extubation. The retention of solids and liquids in the stomach has been described during endoscopic procedures, and there are also increasing reports of gastric content retention detected by perioperative gastric ultrasound [70].

Together, it seems that treatment with GLP-1 and GIP/GLP-1 receptor agonists per se could have a protective role in respiratory infections or at least in favorable outcomes. However, on the other hand, treatment with GLP-1 and GIP/GLP-1 receptor agonists is also associated with an increased risk of gastric content retention and aspiration or regurgitation in the perioperative period. While considering the benefits of GLP-1 and GIP/GLP-1 receptor agonist therapy, caution is needed in clinical scenarios for discontinuation of drugs before procedures, as people with type 2 diabetes risk worsening diabetes control. A compromise should be accepted to bring about the greatest benefit with the least acceptable cost.

### 3.4. Obstructive Sleep Apnea

Obstructive sleep apnea is marked by episodes of apnea and hypopnea during sleep, leading to hypoxemia, hypercapnia, and frequent awakening. These effects result in significant drowsiness during the day. The condition affects an estimated 900 million people worldwide. It is also a risk factor for cardiovascular disease. Randomized control trials have not shown that continuous positive airway pressure (CPAP) therapy reduces the rate of cardiovascular events or mortality in this population. High body fat or adiposity is well known to be associated with obstructive sleep apnea and its related problems, and the advantages of weight loss have been recognized for a long time [71,72]. Furthermore, there also appears to be a link between the severity of obstructive sleep apnea and the reduced native GLP-1 response, which further deteriorates glucose metabolism, causes weight gain, and increases the risk of developing type 2 diabetes. Therefore, the GLP-1 and GIP/GLP-1 receptor agonists in this clinical setting have value in their weight reduction potential and glucose metabolism normalization as well as cardiovascular risk reduction and could even progress to first-line therapy for obstructive sleep apnea [73].

Liraglutide was evaluated in the SCALE sleep apnea randomized trial in non-diabetic patients. Patients with obesity and moderate-to-severe sleep apnea defined by the apnea-hypopnea index (AHI) ≥ 15 events per hour but unwilling to use or incapable of using CPAP therapy were randomized to receive either liraglutide 3.0 mg daily or placebo in addition to diet and exercise. After 32 weeks, the reduction in AHI was significant in the group treated with liraglutide, that is, for −12.2 events per hour compared to −6.1 events per hour in the control group, in addition to significant weight loss. Post hoc analysis revealed a significant association between weight loss and all end points of obstructive sleep apnea. Cardiometabolic parameters also improved in the group treated with liraglutide. The safety profile of liraglutide was acceptable [74]. A two-center randomized controlled study with liraglutide was conducted in people with type 2 diabetes and obstructive sleep apnea requiring CPAP. After a 3 month intervention period, in people receiving liraglutide on top of CPAP therapy, after significant weight loss, AHI significantly decreased from 31.0 ± 7.3 to 26.1 ± 7.1 events per hour, while and mean systolic blood pressure were all significantly reduced compared with the control group. The minimum oxygen saturation significantly increased from 80.3 ± 5.8% to 83.4 ± 5.8% in the liraglutide treated group compared to unsignificant changes in the placebo-controlled group only treated with CPAP. Cardiometabolic parameters also improved significantly. This was achieved without an unreasonable number of side effects [75]. An ongoing open-label prospective study is evaluating the effect of liraglutide (1.8 mg once a day) alone, liraglutide 1.8 mg daily with CPAP, CPAP alone, or without treatment in people with obstructive sleep apnea, obesity, and type 2 diabetes. This study is designed to examine the impact of weight loss-induced reductions in AHI, as well as secondary cardiometabolic parameters. It will be the first study to compare GLP-1 receptor agonist therapy and CPAP efficiency head-to-head [76].

In a recently published randomized controlled trial, SURMOUNT-OSA, tirzepatide was evaluated at maximum tolerated doses of 10 and 15 mg a week compared to placebo for its efficacy in reducing AHI in adults with obesity and moderate to severe obstructive sleep apnea. SURMOUNT-OSA was composed of two trials. The first trial enrolled participants who did not use CPAP therapy, while the second trial enrolled CPAP users at baseline. At inclusion in the study, the mean AHI of the participants in the first trial was 51.5 events per hour, while in the second trial it was 49.5 events per hour. After 52 weeks, tirzepatide significantly reduced the primary endpoint in both trials, reducing AHI by a mean of −25.3 in the first (−5.3 for placebo) and −29.3 (−5.5 for placebo) events per hour in the second trial. This was accompanied by significant body weight, hypoxic burden, high-sensitivity C-reactive protein (hsCRP), and reduction in systolic blood pressure, as well as improved sleep-related patient-reported outcomes. The safety profile of tirzepatide was in line with previous trials. The authors conclude that this result is clinically relevant, as a 50% reduction in AHI was proposed as clinically relevant [77]. Hypoxic burden is more strongly associated with cardiovascular risk than AHI. The results of SURMOUNT-OSA could, based on the reduction of hypoxic burden, translate into a reduction in cardiovascular risk. Furthermore, this reduction could be underestimated, since tirzepatide resulted in a reduction in a wide range of cardiometabolic risk factors, probably leading to even a more extensive reduction in cardiovascular risk [78].

It has also been shown that in obstructive sleep apnea models, both in vitro and in vivo, liraglutide enhanced the Nrf2 and inhibited mitogen-activated protein kinase (MAPK)/NF-κB signaling pathways, leading to alleviation of chronic intermittent hypoxia induced injury associated with cognitive defects [79].

Although we mention only a few of the most prominent clinical trials, liraglutide and tirzepatide show consistent efficacy of GLP-1 and GIP/GLP-1 receptor agonists in obstructive sleep apnea settings. These effects seem to be primarily driven by weight loss in addition to reduction in cardiometabolic risk. Unfortunately, these studies have a limited duration, so long-term efficacy in weight control, end points of obstructive sleep apnea, and cardiovascular risk remain to be seen. Adherence to therapy with respect to the safety profile is also to be considered.

### 3.5. Pulmonary Hypertension

Pulmonary hypertension is an entity composed of a multifactorial group of disorders that have in common an elevated mean pulmonary artery pressure (mPAP) of >20 mmHg at rest. According to etiology, it is classified into five groups: group 1—pulmonary arterial hypertension due to vascular remodeling of the pulmonary arteries, group 2—pulmonary hypertension associated with left-sided heart failure, group 3—pulmonary hypertension associated with lung disease, group 4—pulmonary hypertension associated with pulmonary artery obstructions, and group 5—pulmonary hypertension with unclear and/or multifactorial mechanisms. Currently, it is estimated to affect approximately 1% of the world’s population [80]. Group 2 is the most common. Therefore, the most pronounced effect of GLP-1 and GIP/GLP-1 receptor agonists could be expected in people with heart failure with preserved ejection fraction who have already been shown to benefit from these drugs. In a recent meta-analysis, in patients with heart failure with mildly reduced and preserved ejection fraction, most of whom had overweight or obesity, form STEP-HFpEF, STEP-HFpEF DM, and patients with investigator-reported history of heart failure with mildly reduced and preserved ejection fraction form SELECT and FLOW trials with semaglutide 2.4 mg in the first 3 and 1.0 mg in the last trial, semaglutide reduced the combined endpoint of cardiovascular death or worsening of heart failure events combined (hazard ratio 0.69, 95% CI: 0.53–0.89) and worsening heart failure alone (hazard ratio 0.59 95% CI: 0.41–0.82) compared to placebo, while there was no significant effect on reduction of cardiovascular death alone [81]. The recently published SUMMIT trial investigated the effects of tirzepatide treatment in individuals with obesity and heart failure with a preserved ejection fraction of at least 50%. The results demonstrated a relative risk reduction of 38% in the composite outcome of death from cardiovascular causes or worsening of heart failure events in participants treated with tirzepatide compared to those who received a placebo after a median follow-up period of 104 weeks. This reduction was accompanied by improved functional results. However, tirzepatide did not lead to a reduction in cardiovascular deaths alone, in line with the effects observed with semaglutide [82]. There was no report of pulmonary hypertension in these trials. As for the other groups of pulmonary hypertension, this remains to be seen. Data are scarce, and are mainly found in non-clinical models.

In non-diabetic rats with flow-induced pulmonary hypertension (monocrotaline injection followed by aorto-caval fistula), a 3-week treatment with exendin-4 significantly reduced right ventricle mass and pulmonary artery pressure, thus improving right ventricular function and reducing overall mortality in rats. These effects were achieved through direct vasoactive and anti-inflammatory effects of exendin, as well as modulation of the pulmonary artery smooth muscle cell phenotype. These responses were also associated with the reduction of interleukin-1beta and downstream signaling molecules [83]. Similar findings have been reported for liraglutide that prevented and reversed monocrotaline-induced pulmonary hypertension, right ventricular hypertrophy, and remodeling of the pulmonary artery wall in rats. These effects were mediated by the endothelial nitric oxide synthase (eNOS)/soluble guanylate cyclase/protein kinase G and Rho kinase pathways [84]. This was further explained by showing that liraglutide significantly inhibited platelet-derived BB growth factor proliferation, migration, and motility in smooth muscle cells of the pulmonary artery. These results are mitigated by inhibiting the cellular Drp1/nicotinamide adenine dinucleotide phosphate (NADPH) oxidases (NOX) pathways and Atg-5/Atg-7/Beclin-1/LC3β-dependent autophagy pathways in pulmonary hypertension [85]. Additionally, it was shown that in hypoxia-induced pulmonary hypertension, liraglutide reduced pulmonary artery systolic pressure and right ventricular hypertrophy through the activation of eNOS by normalizing the endothelin B pathway and activation of the AMP-activated protein kinase (AMPK) pathway in mice [86].

Based on non-clinical and clinical data, in 2021, Lee proposed a hypothesis that GLP-1 receptor agonists could be potential therapeutic candidates in pulmonary hypertension patients recovering from COVID-19 by regulating the right ventricular systolic pressure and pulmonary artery remodeling [87]. This hypothesis remains to be confirmed, and no studies have been reported that tested it.

### 3.6. Lung Cancer

According to the WHO, lung cancer was the most commonly diagnosed cancer in 2022 (12.4% of all cancer cases worldwide) and the leading cause of cancer-related death (18.7%) [88]. There is evidence that the incidence of lung cancer increases in people with prediabetes and diabetes [89]. GLP-1 receptor agonists may have a neutral or beneficial effect on lung cancer [90,91,92,93]. In a study by Tabernacky et al., de-identified electronic health records of more than 1,000,000 people with type 2 diabetes were analyzed over a 15-year period. They showed that among patients treated with non-insulin antidiabetic drugs (with the exception of alpha-glucosidase inhibitors), GLP-1 receptor agonists had the lowest risk ratio for lung cancer (0.49, 95% CI: 0.41–0.59) compared to insulin regardless of histological type, gender, race, or smoking status; regrettably, records did not include information on the duration of use or dose of GLP-1 receptor agonists, thereby precluding a thorough analysis of the impact of these variables on the outcomes. [94]. These beneficial effects could be partly explained by the effect of liraglutide in the non-clinical setting. Liraglutide was shown to inhibit lung cancer cell proliferation, cell cycle, and migration compared to control in cell cultures, in addition to inhibiting the epithelial-mesenchymal transition process. There was also evidence that liraglutide suppressed lung cancer cell proliferation in vivo. Furthermore, liraglutide also abrogated high glucose-induced cell senescence, as well as suppressed lung aging and stress of the endoplasmic reticulum [95].

### 3.7. Lung Transplantation

There is no evidence on the effect of incretin-based therapy on lung transplant outcomes. GLP-1 receptor agonists can be used in weight loss strategies prior to lung transplantation [96], as it has been shown that mortality risk increases with BMI > 35 kg/m^2^ and that the magnitude of pre-transplant weight loss is directly correlated with improved post-transplant survival [97]. A small retrospective study evaluated 21 patients with an average BMI of 36.34 kg/m^2^ using GLP-1 receptor agonists to lose weight before lung transplant. They found that 14 (66.7%) of the patients achieved the desired weight loss in this group and lost on average 11.8% of their initial body weight. On the other hand, there was no report on post-transplant outcomes [98]. Considering the tolerability of GLP-1 receptor agonists in the clinical setting after lung transplantation, 58 lung transplant recipients were evaluated, starting GLP-1 receptor agonists (41% semaglutide, 38% dulaglutide) in the median year 2.1 after transplantation. The median duration of GLP-1 receptor agonists therapy was 1.4 years, and 31% of the patients discontinued the drug, 7% due to nausea and 5% due to vomiting. During GLP-1 receptor agonists therapy, 9% of the patients had cell rejection and 14% had lung allograft dysfunction. The median weight loss was 5 kg. They demonstrated the safety and relatively good tolerability of GLP-1 receptor agonists in this clinical circumstance, but the discontinuation was more common than in general population, most probably due to the overlap and multiplication of side effects of lung transplant medications with those of GLP-1 receptor agonists [99].

Management of post-transplant diabetes can be challenging, as immunosuppressive drugs such as glucocorticoids, calcineurin inhibitors, and mTOR (mammalian target of rapamycin) inhibitors are associated with induction of hyperglycemia or worsening of glucose control. Several clinical trials evaluated the use of GLP-1 receptor agonists in the treatment of solid organ post-transplant diabetes. The number of lung transplant patients was minor among the generally small number of patients included. There is consistency in the efficacy and safety of these drugs in the post-solid organ transplant period in both glycemia and weight management. In addition, there is evidence of no interference with immunosuppressive therapy [100,101]. Data show that use of GLP-1 receptor agonists in the post-transplant setting may be beneficial in ameliorating immunosuppression induced beta cells dysfunction as well as positively affecting the immune function in metabolic disorders through suppression of the activation of the CD4+ T lymphocyte cytokine expression [102].

## 4. Future Directions and Possibilities

Based on the data presented, we can establish that the use of GLP-1 and GIP/GLP-1 receptor agonists seems to be safe and beneficial in many respiratory disorders. Despite this, there is undoubtedly ground for further research on the use of GLP-1 and GIP/GLP-1 receptor agonists in the broad field of respiratory disease. Several questions remain to be answered. First, although data show that they beneficially influence metabolic and inflammatory pathways in the lung, mediating both Th2-mediated and non-Th2-mediated inflammatory profiles (Figure 2), the complete mechanism of action in the lungs has not yet been fully elucidated. Second, long-term safety with regard to adipose tissue loss and, in particular, muscle mass loss must be determined, as chronic pulmonary diseases due to continuous inflammation are known to lead to sarcopenia. Third, the optimal dose of these drugs remains to be established; as in clinical scenarios primarily of lung problems, the doses do not need to be the same as for cardiometabolic treatment. Fourth, the respiratory disease patient subpopulations that would benefit the most from the treatment of the GLP-1 and GIP/GLP-1 receptor agonists must be determined. Fifth, according to the present data, most of the studies were retrospective in nature and focused on findings without a primary focus on analyzing the effects of these drugs on lung function; the lung findings were based on secondary or additional analysis. Consequently, prospective studies are necessary to directly assess the impact of GLP-1 and GIP/GLP-1 receptor agonists on the lungs, with primary endpoints focused on lung-related outcomes. Sixth, there is a need for prospective clinical trials that evaluate their use in particular clinical settings, including uncontrolled non-Th2 asthma in obese individuals; influence on respiratory control and upper airway muscle tone, sleep architecture, and quality, and whether they have a potential synergistic effect not only with CPAP, but also with other devices used in the treatment of obstructive sleep apnea treatment; and regulation of right ventricular systolic pressure and remodeling of the pulmonary arteries in pulmonary hypertension, as well as their effects in pulmonary hypertension outside group 2, to name just a few. These future studies must be strictly designed and sufficiently powered to provide as straightforward and clear as possible information on the future use and establishment of GLP-1 and GIP/GLP-1 receptor agonists in daily pulmonary clinical practice. 

## 5. Conclusions

GLP-1 and GIP/GLP-1 receptor agonists constitute a promising opportunity for the treatment of respiratory disorders. Due to their multifaceted beneficial properties, these agents appear to be viable candidates for the treatment of a variety of respiratory conditions in addition to the well-established lung-directed therapies. As research progresses, these agents could become more central in the treatment of respiratory diseases.

## Data Availability

Not applicable.
